# Diagnostic performance of an ultrasensitive HRP2-based malaria rapid diagnostic test kit used in surveys of afebrile people living in Southern Ghana

**DOI:** 10.1186/s12936-021-03665-7

**Published:** 2021-03-02

**Authors:** Festus K. Acquah, Dickson Donu, Evans K. Obboh, Dorcas Bredu, Bernice Mawuli, Jones A. Amponsah, Joseph Quartey, Linda E. Amoah

**Affiliations:** 1 Immunology Department, Noguchi Memorial Institute for Medical Research (NMIMR), University of Ghana, Accra, Ghana; 2 West African Centre for Cell Biology of Infectious Pathogens (WACCBIP), University of Ghana, Accra, Ghana; 3 School of Medical Sciences, University of Cape Coast, Cape Coast, Ghana; 4 Parasitology Department, Noguchi Memorial Institute for Medical Research (NMIMR), University of Ghana, Accra, Ghana

**Keywords:** Ultrasensitive RDT, Sensitivity, Specificity, Asymptomatic

## Abstract

**Background:**

The Alere™ Malaria Ag P.f Ultra-sensitive RDT (UsmRDT) kit is an HRP2-based malaria rapid diagnostic test (RDT) with enhanced sensitivity relative to the SD Bioline Malaria Ag P.f RDT (mRDT) kit. However, the diagnostic performance of the UsmRDT kit has not been evaluated in Ghana.

**Methods:**

A total of 740 afebrile participants aged between 3 and 88 years old were recruited from the Central and Greater Accra Regions of Ghana during the off-peak malaria season. Axillary body temperature was measured, and a volume of 1 ml venous blood was drawn from each participant. Prior to separating the blood into plasma and packed cell pellets via centrifugation, the blood was spotted onto one UsmRDT and one mRDT kit and also used to prepare thick and thin blood smears as well as filter paper blood spots. *Plasmodium falciparum* specific polymerase chain reaction (PCR) was performed on gDNA extracted from 100 µl of the whole blood.

**Results:**

The overall positivity rate for microscopy, PCR, UsmRDT and mRDT kit were 20.4%, 40.8%, 31.3% and 30.8%, respectively. Overall, the UsmRDT identified 9.3% (28/302) more PCR positive samples than the mRDT kits. All samples that were negative by the UsmRDT kit were also negative by the mRDT kit. Overall, the sensitivity and specificity of the UsmRDT was 73% (221/302) and 89% (388/436), respectively, while that for the mRDT kit was 58% and 90%, respectively.

**Conclusion:**

Although the UsmRDT kit was not as sensitive as PCR at detecting asymptomatic *P. falciparum* carriage, it correctly identified *P. falciparum* in 9.3% of the study participants that were not captured by the mRDT kit. In malaria endemic settings, the UsmRDT would provide an added advantage by identifying more asymptomatic *P. falciparum* carriers than the mRDT kit for targeted treatment interventions.

## Background

Global malaria eradication requires all malaria endemic countries to individually be free of all malaria parasites [1]. Although malaria case mortality and morbidity are declining as the global incidence rate per 1000 population at risk has reduced from 71 to 57 from 2010 to 2018, sub-Saharan Africa (SSA) remains highly burdened with malaria with about 24 million cases recorded in children alone in 2018 [2]. Apart from children under the age of 5 years who have not developed immunity to malaria, pregnant women are also a high-risk population as malaria in pregnancy can result in maternal anaemia, low birth weight, spontaneous abortions and other poor birth outcomes [3]. Due to the very high mortality and morbidity of malaria in SSA, the main focus of malaria control interventions in these endemic countries is to reduce the severity and incidence of symptomatic malaria [4, 5].

However, asymptomatic malaria parasite carriage has been reported to be very high in SSA, with a study conducted in the Eastern Region of Ghana reporting a prevalence of asymptomatic participants to be 33.1% by rapid diagnostic tests (RDT) and 66% by molecular tests [6]. Asymptomatic parasite carriers are contributors to malaria transmission as asexual parasites from these infections develop into gametocytes, the transmissible forms of the parasite [7–9]. Asymptomatic infections have been suggested to be a precursor of symptomatic infections [10, 11], making it very important to identify and clear these parasite reservoirs. Furthermore, during pregnancy, malaria parasites have been shown to sequester in the placenta, keeping most of them away from peripheral blood and limiting their subsequent detection by microscopy, the gold standard for detection [3]. The World Health Organization (WHO) recommends prompt diagnosis of malaria infection in pregnant women to circumvent the manifestations of pregnancy associated malaria [2]. Sensitive and effective point of care devices including ultra-sensitive RDT kits can enhance the early detection of low-density malaria parasites in pregnant women, which would ultimately result in prompt treatment.

Asymptomatic parasitaemia is predominantly at densities below the detection limit of microscopy. These submicroscopic densities of parasites require enhanced detection tools, such as polymerase chain reaction (PCR) for detection. A variety of PCR techniques have been developed for the detection of malaria parasites. Advantages of PCR include the ability to detect parasite densities as low as 0.5 parasites per microlitre (p/µl) for real time PCR [12] and about 5 p/µl for nested PCR [13]. The main disadvantages of PCR are that it is not a rapid test and requires highly specialized instrumentation and skilled persons to perform the tests [14].

Malaria RDTs were developed to enhance the diagnosis of malaria by providing rapid point of care diagnosis [15]. The analytical limit of detection of the standard malaria RDT kit is 800 pg/ml of HRP2 antigen in whole blood [16], with the sensitivity reducing as parasite density reduces to below 200 p/µl [17]. The most sensitive malaria RDT kit detects the HRP2 antigen, which is specific to *Plasmodium falciparum*, the *Plasmodium* species which accounts for most (over 95%) of all malaria infections in SSA [2]. An improvement to the standard HRP2 based malaria RDT, the UsmRDT kits, which have an analytical detection limit of 80 pg/ml of HRP2 antigen [18] were introduced in 2017, with the main aim of enhancing the rapid detection of low density asymptomatic malaria parasite carriers. One main disadvantage of malaria RDT kits is that they provide indirect evidence of parasite prevalence, with the HRP2 based test kits detecting HRP2 antigen that have persisted after a recently cleared infection [19–21]. Among febrile children under 5 years, the UsmRDT has been reported to have a slightly higher sensitivity (75%) than the Abbott combo-RDT kit (73%) [22]. A previous study in pregnant women from a moderate malaria transmission site in Colombia identified a slightly higher sensitivity of the UsmRDT over the standard RDT (85.7% versus 82.8%), but similar specificity (> 99.0%) [23]. Using archived blood samples from pregnant women from a low transmission setting in Indonesia, a study reported no difference in performance of the UsmRDT relative to the standard CareStart combo-RDT kit [24]. These contrasting reports present the need to provide more evidence on the performance of the UsmRDT against standard malaria RDT kits to influence policies relating to malaria diagnostics.

This study set out to determine the performance of the UsmRDT kit at diagnosing asymptomatic malaria parasite carriage by afebrile children and adults in two communities where parasite carriage is high and pregnant women from a third community where parasite carriage is low in Ghana during the off-peak malaria season.

## Methods

### Study design, site and sampling

**Fig. 1:**
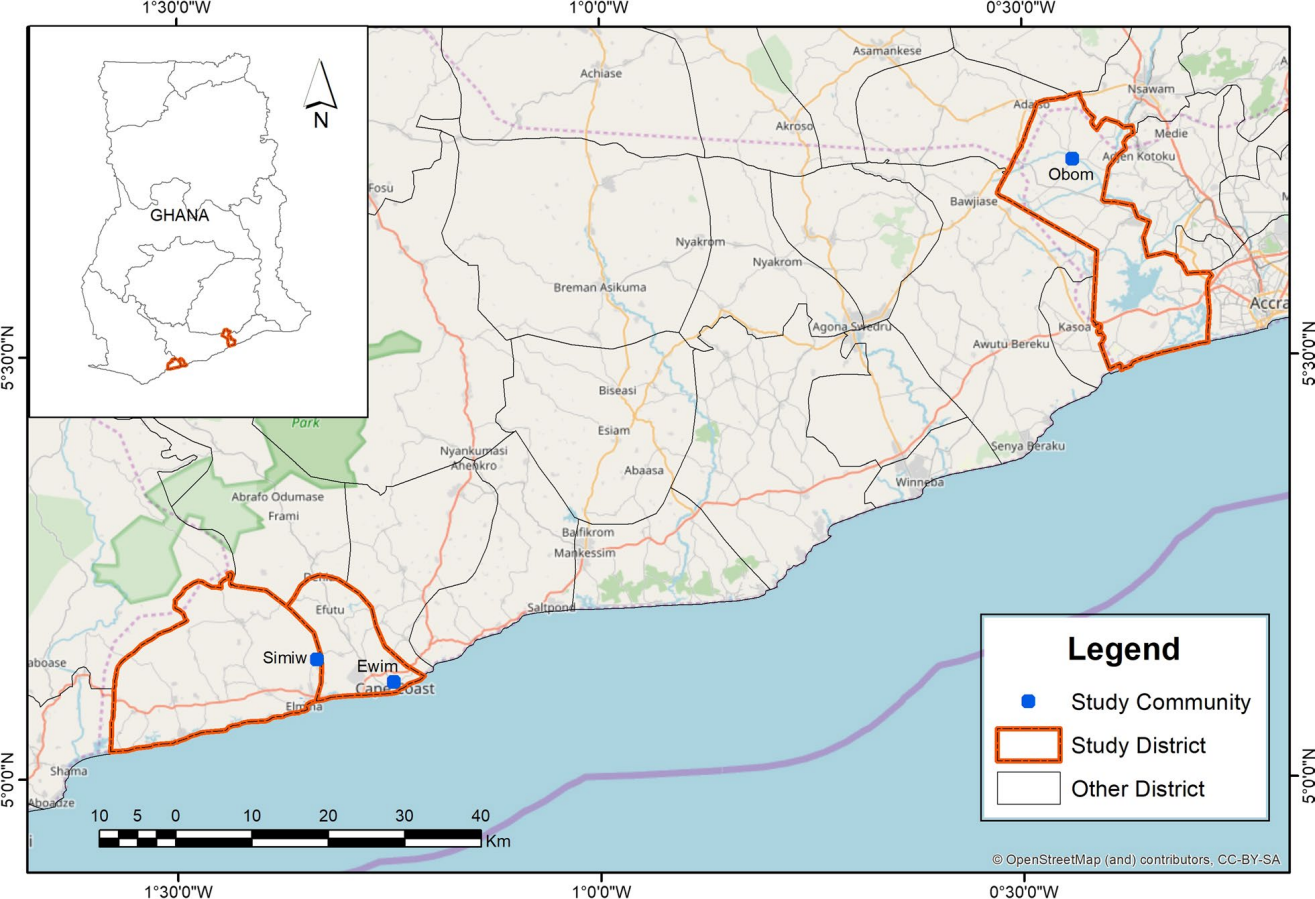
A map of Ghana highlighting the study sites. The map was created by Mr. Richard Adade, GIS & Remote Sensing Unit, Department of Fisheries using shapefiles from the Survey Department of the Ghana Statistical Services and ArcMap GIS v10.5

This study was a pilot cross sectional study conducted between December 2019 and January 2020 in three communities in southern Ghana (Fig. 1). Recruitment from Obom (N = 292) and Simiw (N = 348) was community based while pregnant women were recruited from the Ewim Polyclinic (hospital based, N = 100). All volunteers aged 3 years old and above without visible symptoms of malaria and resident in Obom or Simiw during the sample collection period were eligible to be enrolled into the study. If any volunteer was febrile or exhibited any obvious signs and symptoms of malaria, they were not enrolled into the study but rather referred to the nearest health facility. All pregnant women attending antenatal care at the Ewim Polyclinic regardless of age of pregnancy or mother were also eligible to enrol into the study. Consecutive sampling was used to enrol a total of 740 participants of which a 100 were pregnant women attending an antenatal clinic in Ewim. Written informed consent was obtained from all the recruited study participants.

Obom is a peri-urban community in the Ga South municipality of the Greater Accra Region with high malaria parasite prevalence of 86% in the rainy season [8]. Ewim is a sub-urban community in the Cape-Coast municipality of the Central Region with low malaria parasite prevalence (4.9% in the rainy season) [25]. Simiw is a rural community in the Komenda Edina Eguafo Abrem District (KEEA) of the Central Region with high malaria parasite prevalence (87.5% in the rainy season) [25]. Malaria transmission in southern Ghana is predominantly perennial, with the peak malaria season occurring in June and July.

### Sample collection and processing

The axillary temperature of each participant was measured and recorded prior to sample collection. Venous whole blood (1 ml) was collected from each participant into EDTA vacutainer^**®**^ tubes. Thick and thin blood films were prepared and processed using standard protocols [26]. Four 50 µl drops of blood were spotted onto Whatman^**®**^ #3 filter paper (BD, UK). The SD Bioline Malaria Ag P.f RDT kit (mRDT: 05FK50-40, Abbott, USA) and the ultra-sensitive Alere™ Malaria Ag P.f RDT (UsmRDT: 05FK140, Abbott, USA) were both used to test for the presence of HRP2 antigen as a proxy for the presence of *P. falciparum* according to manufacturer’s instructions. Briefly, both RDTs had their sample wells spotted with whole blood using the inverted cups supplied with the RDT package. Three drops of every kit’s accompanying assay diluent were dropped in the appropriate well immediately. The results of the RDT kits were read 15 min and 20 min after assay diluent addition for the mRDT and UsmRDT kit, respectively. All participants in this study were informed to visit the nearest health facility as soon as they develop any malaria related symptom. Thick and thin blood smears were prepared and stained according to standard protocols for identification and quantification of *P. falciparum* [26]. A 100 µl aliquot of whole blood was used for genomic DNA extraction and the rest was separated into plasma and packed blood cell pellets and individually stored at − 20 °C.

### *Plasmodium* parasite density estimation

Two independent certified malaria microscopists read the thick and thin blood smears. All discordant results were settled by an independent reading of the respective slides by a third microscopist. Parasite density was calculated based on the assumption that there are 8000 white blood cells (WBCs) in one microlitre of blood as the number of parasites observed per 200 white blood cells multiplied by 40. A slide was regarded as negative when no parasite was identified within a minimum of 200 fields.

### Molecular detection of *P. falciparum* by PCR

Genomic DNA was extracted from all the 740 samples using the Zymo^®^ DNA Kit (Zymo Research, Irvine, U.S.A) for whole blood samples according to manufacturer’s instructions. Briefly, 100 µl of whole blood was lysed with 400 µl of lysis buffer prior to running over the spin column. The DNA was eluted using 100 µl of elution buffer and stored at 4 °C for immediate use. The *P. falciparum 18S rRNA* gene was amplified from 20–40 ng of the extracted DNA in a 15 µl reaction volume. The PCR reactions were made up of 2.5 mM MgCl_2_, 200 nM deoxyribonucleoside triphosphate mix (dNTPs), 1 U of Onetaq™ DNA polymerase (NEB, UK) and 250 nM each of forward and reverse primers (rPLU5 and rPLU1) for the primary reaction and rFAL1 (F) and rFAL2 (R) for the nested reaction [27]. Genomic DNA from the 3D7 strain of *P. falciparum* (MRA 102G) and double-distilled water were used as positive and negative control samples respectively for the PCR amplifications. The PCR amplification products were resolved on 2.0% agarose gels stained with 0.5 µg/ml ethidium bromide. After electrophoresis, the gels were visualized under UV and the image captured using a Vilber Lourmat Gel Dock System^®^ (Vilber, Wielandstrasse, Germany).

### Data analysis

After processing of the blood smears according to standard procedures [28], a sample was determined as negative for malaria parasites by microscopy if no parasites were observed in 200 fields, otherwise, the sample was classified as microscopy positive.

The demographic and haematological data of all the participants from the three sites were compared using Kruskal–Wallis analysis and Dunn’s multiple comparison test was used as post hoc test to assess any observed differences between groups in GraphPad Prism version 5. Independent students *t* test was used to compare continuous numerical data from pregnant women and non-pregnant women in GraphPad Prism version 5. Linear correlation analysis was also used to assess possible associations between parasite density, age, haemoglobin levels and axillary temperature.

The sensitivity and specificity as well as the positive and negative predictive values were determined using IBM SPSS version 21. IBM SPSS version 21 was also used to determine the level of agreement between the different tests were determined using the Cohen’s kappa inter-rater reliability test.

## Results

### Participant demographic details

Children aged between 3 and 17 years old and females represented 57.70% (427/740) and 67.20% (497/740) of the total study population respectively. The median age of the afebrile pregnant women from Ewim was significantly higher than participants from Obom and Simiw (Kruskal–wallis H = 67.66, p < 0.0001), whose median ages were not significantly different. The overall median axillary temperature for all the three study cohorts was about 36.6 °C (IQR: 36.20–36.90) (Table 1). Parasite density of participants from Simiw and Obom did not significantly correlate with either haemoglobin levels or age (Additional file 1). Haemoglobin levels were moderately and positively correlated with age in both sites (r = 0.515, p < 0.001 and r = 0.458, p < 0.001 for Simiw and Obom, respectively).

**Table 1:** Demographic details of study participants

Cohort	Ewim (100)	Obom (292)	Simiw (348)	p-value^a^	Pregnant women	Non-pregnant women	p-value^b^	Total (740)
Age (yrs)	27.0 (22.0–32.0)	14.0 (10.0–23.0)	14.0 (11.0–32.0)	< 0.01	27.00 (23.00–32.00)	29.00 (24.00–34.00)	0.07	15.0 (11.0–30.0)
HB (g/dl)	10.6 (9.6–11.2)	12.2 (11.1–13.9)	11.2 (10.3–12.5)	< 0.0001	10.55(9.53–11.20)	12.45 (11.30-13.40)	< 0.0001	11.4 (10.5–12.8)
Temp (^o^C)	36.2 (35.9–36.5)	36.8 (34.5–36.9)	36.6 (32.0–38.0)	< 0.01	36.30 (35.90–36.58)	36.60 (36.10–36.87)	0.001	36.6 (3.2–36.9)
PCR								
Prevalence % (n/N)	13.0 (13/100)	39.3 (115/292)	50.0 (174/348)	< 0.001	10.54% (13/96)	27.63% (21/76)		40.8 (302/740)
Microscopy								
Prevalence % (n/N)	6.0 (6/100)	23.3 (68/292)	22.1 (77/348)	< 0.001	6.25% (6/96)	9.21% (7/76)		20.4 (151/740)
UsmRDT								
Prevalence % (n/N)	7.0 (7/100)	41.7 (121/290)	39.6 (138/348)	< 0.001	7.29% (7/96)	18.42% (14/76)		31.3 (231/738)
mRDT								
Prevalence %(n/N)	7.0 (7/100)	35.3 (103/292)	33.9 (118/348)	< 0.001	7.29% (7/96)	14.42% (11/76)		30.8 (228/740)
PD (p/µl)	4882.0 (2591.0–14,324.0)^#^	280.0 (129.3–625.5)	471.0 (139.5–2065.0)	< 0.001	4882.0 (2591.0–14,324.0)^#^	200.0 (80.0–840.0)^*^	ND	400.0 (160.0–1154.0)

HB: haemoglobin level; Temp: temperature; PD: parasite density; p: parasites. mRDT: SD Bioline Malaria Ag P.f RDT kit; UsmRDT, ultra-sensitive Alere™ Malaria Ag P.f RDT; ND: not done due to small sample numbers

^#^Values obtained from only 6 samples

*Values obtained from only 7 samples; yrs, years

^a^p is the p value for comparisons between Ewim, Simiw and Obom

^b^p is for comparisons between pregnant and non-pregnant women aged between 18 and 40 years old. Data from 4 pregnant women who were not within the 18 and 40 years old age range were excluded from the analysis of pregnant women. The values in the table are presented as medians (interquartile ranges)

Since there was no significant difference (Mann–Whitney U = 3059, p = 0.07) between the ages of non-pregnant women who were 18 to 40 years old from Simiw and Obom, both of which are high prevalence settings, their data was combined for comparison with similarly aged pregnant women of from Ewim. Haemoglobin levels and axillary temperatures of pregnant women were significantly lower than non-pregnant women of the same age group (Mann–Whitney U = 1186, p < 0.0001 and Mann–Whitney U = 2611, p = 0.001 respectively) (Table 1).

### Parasite detection by different diagnostic techniques

Overall, 40.8% (302/740), 20.4 (151/740), 31.3 (231/738) and 30.8 (228/740) of participants were positive by PCR, Microscopy, UsmRDT and mRDT, respectively (Table 1). By site, parasite prevalence estimated by PCR was 13.00% (13/100), 39.38% (115/292) and 50.00% (174/348) in Ewim (pregnant women), Obom and Simiw respectively and represented the highest prevalence estimated among the four diagnostic tools used. Microscopy gave the lowest prevalence of 6.00% (6/100), 23.29% (68/292) and 22.13% (77/348) for Ewim, Obom and Simiw, respectively (Table 1).

**Fig. 2:**
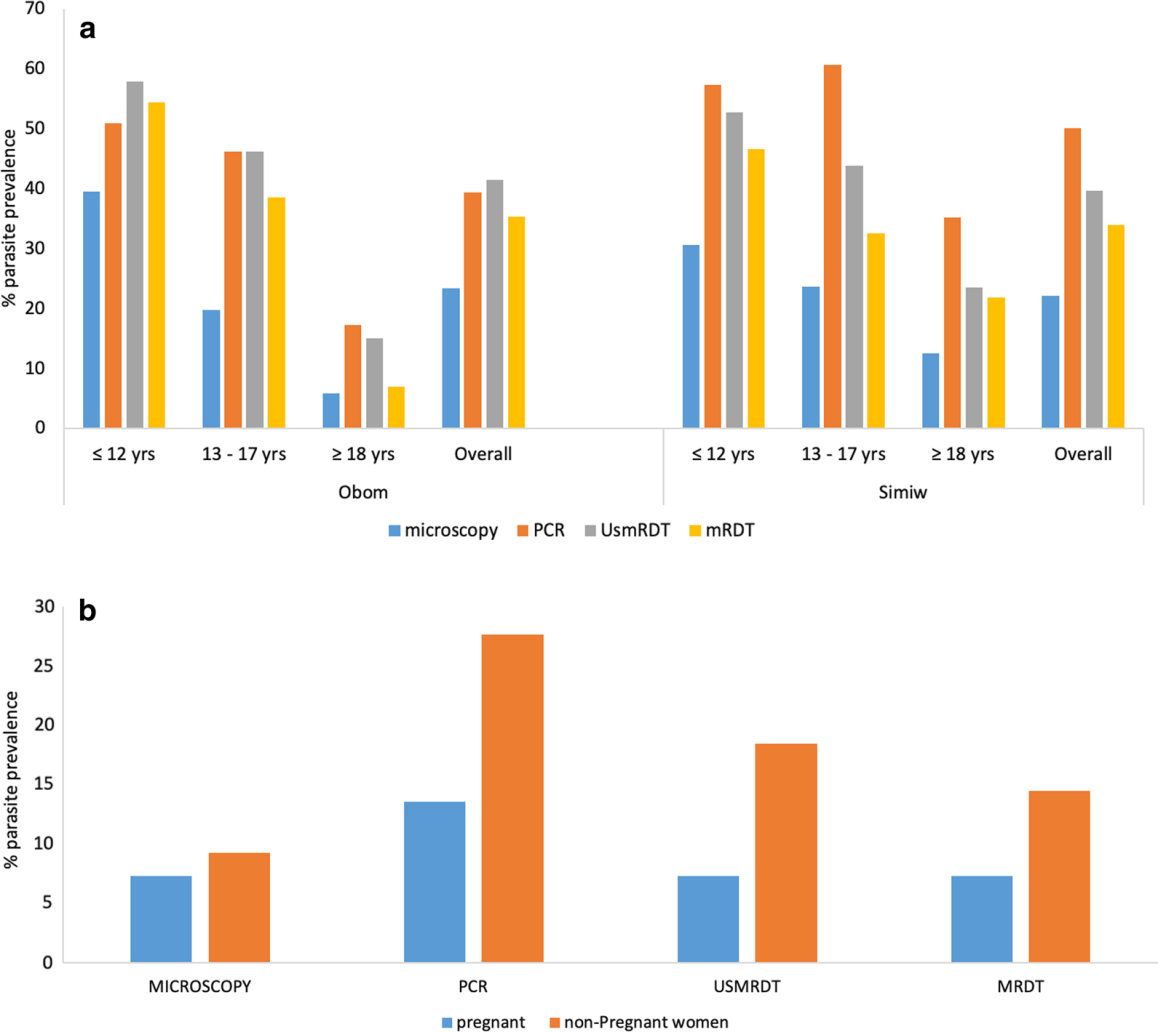
Prevalence of asymptomatic individuals. **a** Age categorized prevalence of *P. falciparum* in Obom and Simiw by the different diagnostic methods. **b** Prevalence of *P. falciparum* prevalence in pregnant women from Ewim and non-pregnant women of the same age group from Simiw and Obom (combined) by the different diagnostic tests. PCR: *P. falciparum* species PCR; micro: microscopy; UsmRDT: ultra-sensitive Alere™ Malaria Ag P.f RDT kit and mRDT, SD Bioline Malaria Ag P.f RDT kit

The UsmRDT and mRDT kits presented similar parasite prevalence of 7.00% (7/100) among the pregnant women of Ewim, whereas among the participants (male and non-pregnant females) from Obom and Simiw, the UsmRDT recorded a higher prevalence than the mRDT kit in both Obom and Simiw (41.72% (121/290) versus 35.3% (103/292) and 39.62% (138/348) versus 33.91% (118/348), respectively) (Fig. 2a). Two samples from Obom had missing UsmRDT results and were excluded from the analysis.

Categorizing the study participants from Obom and Simiw into younger children (12 years old and below), older children (13–17 years old) and adults (18 years old and above) showed a general inverse relationship of age group with parasite prevalence; with adults having the lowest prevalence of *P. falciparum* while children had the highest prevalence of the parasite (Fig. 2a). The highest occurrence of false positive (PCR negative, RDT positive) results for both the UsmRDT and the mRDT kits were more prevalent in children from Obom (Fig. 2a).

Parasite prevalence in the pregnant women aged between 18 and 40 years old was lower than similarly aged non-pregnant women for all the different diagnostic tools assed in this study (Fig. 2b). Among the pregnant women, PCR had the highest estimate (10.54% (13/96)), followed by an equal prevalence of UsmRDT and mRDT (7.29% (7/96)) then microscopy (6.25% (6/96)) (Table 1). Among the non-pregnant women PCR similarly gave the highest prevalence (27.63% (21/76)), followed by the UsmRDT (18.42% (14/76)), mRDT (14.42% (11/76)) and finally microscopy (9.21% (7/76)) (Table 1).

Overall, the UsmRDT was able to detect 95.23% (100/105) of all the samples that had parasite densities of ≥ 200 p/µl whilst the mRDT kit detected 88.57% (93/105) of these samples (Tables 3, 4). The sensitivity of the UsmRDT as well as the mRDT reduced to 86.96% (40/46) and 82.61% (38/46), respectively, when the parasite densities of the samples reduced below 200 p/µl (Table 2).

**Table 2:** Sensitivity of the RDT kits at high (≥ 200) and low (< 200) parasite densities

		Simiw	Obom	Ewim	Total
PD ≥ 200 p/μl	UsmRDT	94.34 (50/53)	100.00 (46/46)	66.67 (4/6)	95.23 (100/105)
	mRDT	84.91 (45/53)	95.65 (44/46)	66.67 (4/6)	88.57 (93/105)
PD < 200 p/μl	UsmRDT	83.33 (20/24)	90.91 (20/22)	NA	86.96 (40/46)
	mRDT	83.33 (20/24)	81.82 (18/22)	NA	82.61 (38/46)

PD: parasite density; mRDT: SD Bioline Malaria Ag P.f RDT kit; UsmRDT: ultra-sensitive Alere™ Malaria Ag P.f RDT; NA: none of the samples had parasite densities less than 200 p/μl

### Diagnostic properties of the mRDT and UsmRDT kits

Using PCR as the reference diagnostic tool, UsmRDT had an overall sensitivity and specificity of 73% (220/302) and 89% (389/435), respectively. The mRDT kit had sensitivity and specificity values of 65% (197/301) and 92% (404/438), respectively (Table 3). The UsmRDT and the mRDT had the same sensitivity (53.8(7/13)) and specificity (100 (83/83)) among the pregnant women. In the non-pregnant women, sensitivity was higher for UsmRDT (52.4 (11/24) *versus* 42.9(9/21)) than the mRDT but s specificity was slightly higher for mRDT (96.4(53/55) versus 94.5 (52/55)) (Table 4).

**Table 3:** Diagnostic properties of the UsmRDT and the mRDT kits

	UsmRDT value	95% CI	mRDT value	95% CI
		(Lower CI–Upper CI)		Lower CI–Upper CI
Simiw				
Sensitivity	67.24 (117/174)	59.66–74.04	59.77 (104/174)	44.77–67.07
Specificity	87.86 (152/173)	81.82–92.16	91.91 (159/173)	86.53–95.34
PPV	84.78 (117/138)	77.45–90.12	88.13 (104/128)	80.57–93.12
NPV	72.72 (152/209)	66.01–78.53	69.43 (159/229)	62.96–75.24
Obom				
Sensitivity	83.48 (96/115)	75.15–89.51	72.81 (83/114)	63.53–80.51
Specificity	85.71 (150/175)	79.44–90.37	88.57 (155/175)	82.68–92.71
PPV	79.34 (96/121)	70.83–85.95	80.58 (83/103)	71.37–87.46
NPV	88.76 (150/169)	82.77–92.92	83.33 (155/184)	77.02–88.23
Ewim				
Sensitivity	53.84 (7/13)	26.12–79.60	53.84 (7/13)	26.12–79.60
Specificity	100.00 (87/87)	94.73–100.00	100.00 (87/87)	94.73–100.00
PPV	100.00 (7/7)	56.09–100.00	100.00 (7/7)	56.09–100.00
NPV	93.55 (87/93)	85.95–97.35	93.55 (87/93)	85.95–97.35
Total				
Sensitivity	72.85 (220/302)	67.40–77.71	64.8 (197/301)	59.11–70.11
Specificity	89.43 (389/435)	86.06–92.08	92.18 (401/435)	89.14–94.45
PPV	82.71 (220/266)	77.50–86.94	85.28 (197/231)	79.90–89.46
NPV	82.59 (389/471)	78.79–85.84	78.94 (401/508)	75.08–82.35

NPV: negative predictive value; PPV: positive predictive value; mRDT: SD Bioline Malaria Ag P.f RDT kit; UsmRDT: ultra-sensitive Alere™ Malaria Ag P.f RDT; 95% CI: 95% confidence interval. The data represents values obtained after setting results obtained from PCR as the reference diagnostic tool

**Table 4:** Diagnostic properties of the UsmRDT and the mRDT kits in pregnant women (18 and 40 years old) and similarly aged non-pregnant women

	Pregnant women		Non-pregnant women	
	UsmRDT	mRDT	UsmRDT	mRDT
Sensitivity	53.8 (7/13)	53.8 (7/13)	52.4 (11/24)	42.9 (9/21)
Specificity	100 (83/83)	100 (83/83)	94.5 (52/55)	96.4 (53/55)

mRDT: SD Bioline Malaria Ag P.f RDT kit; UsmRDT: ultra-sensitive Alere™ Malaria Ag P.f RDT. The data represents values obtained after setting results obtained from PCR as the reference diagnostic tool

The level of agreement between both the mRDT kit and the UsmRDT kit and PCR was higher than 0.5 with significant p-values (< 0.05) in all three sites. Overall, the level of agreement between PCR and the RDT kit was higher for UsmRDT (Kappa = 0.63, p < 0.001) than mRDT (Kappa = 0.59, p < 0.001) (Table 5).

**Table 5:** Level of agreement between RDT kits and PCR (PCR prevalence = 40.8%)

	UsmRDT		mRDT	
	Kappa	p-value	Kappa	p-value
Ewim	0.670	< 0.001	0.670	< 0.001
Obom	0.687	< 0.001	0.625	< 0.001
Simiw	0.552	< 0.001	0.517	< 0.001
Total	0.635	< 0.001	0.589	< 0.001

UsmRDT: ultra-sensitive Alere™ Malaria Ag P.f RDT; mRDT: SD Bioline Malaria Ag P.f RDT. Values reported are based on PCR results as the reference category

### Comparison of the mRDT and UsmRDT kits

The level of agreement of the UsmRDT and the mRDT was very high and ranged from a Kappa of 1 in Ewim to 0.84 in Simiw and a Kappa of 0.87 for all the samples tested. Two samples from Obom with missing RDT values were excluded from the analysis (Additional file 1).

**Fig. 3:**
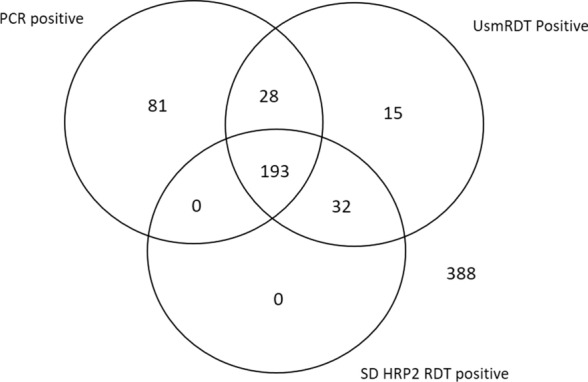
Detection of *P. falciparum* by different diagnostic methods. All samples were grouped into sets belonging to PCR, UsmRDT (ultra-sensitive Alere™ Malaria Ag P.f RDT kit) and mRDT (SD Bioline Malaria Ag P.f RDT kit positive samples)

In total, all the samples that tested negative by UsmRDT kit also tested negative by mRDT kit. However, 43 samples that tested negative by the mRDT kit tested positive by UsmRDT, 65.12% (28/43) of these mRDT negative/UsmRDT positive samples were identified to contain *P. falciparum* parasites by PCR (Fig. 3).

## Discussion

Malaria parasite carriage is predominantly detected using either a malaria RDT or a combination of RDT and microscopy [29]. However both microscopy and malaria RDTs have been found to underestimate parasite prevalence due to their inability to detect low density infections [30]. In this study, we utilized the ultra-sensitive Alere™ Malaria Ag P.f RDT (UsmRDT) kit in addition to the SD Bioline Malaria Ag P.f RDT (mRDT) kit, microscopy and PCR of the *P. falciparum* 18S rRNA gene to identify malaria parasite prevalence in afebrile study participants living in the south of Ghana. This study included pregnant women and children under the age of 10 years old as a high percentage of malaria-related deaths occur in these high risk population [31], and early detection and clearance of malaria parasites at very low levels, detectable by the UsmRDT would obviate these at-risk populations from the catastrophic effects of severe malaria in children and pregnancy associated malaria in pregnant women. Children and pregnant women living in low transmission settings have the added disadvantage of possessing lower immunity against malaria and are likely to experience more severe manifestations of disease than their counterparts living in high transmission settings [32]. Pregnant women from high transmission settings with high levels of immunity often unknowingly harbor malaria parasites in asymptomatic infections, which can alter the outcome of the pregnancy [32]. This makes enhanced and rapid diagnosis of malaria very crucial in both high and low transmission settings.

Microscopy proved to be the least sensitive diagnostic tool in all the three cohorts. This is not surprising as a high proportion of asymptomatic infections are at submicroscopic densities [33]. The observed reduction in the ability of both the mRDT kit and the UsmRDT kit to detect samples with low density parasites was anticipated as the sensitivity of RDT kits are known to decrease with a decrease in parasite density [34]. The UsmRDT kit, which has been reported to have a ten times lower limit of detection compared to the mRDT kit [35] was able to detect a higher number of both high (≥ 200 p/μl) and low (< 200 p/μl) parasite density samples than the mRDT kit, suggesting that the superiority of the UsmRDT kit in this study was not dependent on parasite density.

*Plasmodium falciparum* species-specific PCR detected the highest number of asymptomatic malaria parasite carriers amongst the study participants attending antenatal checkup at Ewim (pregnant women) and from the Simiw community. This result is to be expected as PCR is known to have a lower limit of detection than the UsmRDT, the mRDT and microscopy. During pregnancy, malaria parasites are known to sequester in the placenta, making them unavailable for detection in the peripheral blood by less sensitive techniques such as microscopy [3]. The HRP2 produced by the parasites however enter into circulation and are detectable by RDT kits. Parasite prevalence among non-pregnant women was high than the pregnant women. Although this could be attributable to the fact that the pregnant women are in a low prevalence area, it could also be attributable to the WHO Intermittent Preventive Treatment in Pregnancy (IPTp) policy that involves giving pregnant women sulfadoxine-pyrimethamine to clear any malaria parasites that may be present [36, 37]. In Obom however, the UsmRDT detected a slightly higher parasite prevalence than PCR and a possible explanation for this observation could be the persistence of HRP2 antigen for a period of time after clearance of parasitaemia, resulting in a false positive result (negative by PCR but positive by the RDT). False positive RDT results for HRP2 based malaria RDT kits are not unusual, as the HRP2 antigen is known to persist days to weeks after an infection is cleared [38].

Although false positive test results were recorded for both the mRDT and the UsmRDT, the latter recorded about 4% more than the mRDT kit. False positive results were observed to be high in Obom and in children compared to adults. This has been observed in other studies [20, 21] especially in children recently treated for malaria and this has been attributed to less developed ability of the adaptive immune system of the children in clearing the antigen compared to adults [21]. The higher numbers of false positives detected by the UsmRDT relative to the mRDT is due to the enhanced sensitivity of the UsmRDT at detecting low concentrations of HRP2 antigens [16].

Obom has been reported as a high malaria transmission intensity setting for the past 5 years [27, 39, 40]. However, in this study there was a decline in malaria parasite prevalence detected by both mRDT and PCR, which may be due to successful malaria control interventions that have cleared the parasite.

Overall, the UsmRDT had a slightly higher sensitivity but lower specificity than the mRDT. Also, the performances of both RDTs were better in the high transmission settings than the low transmission settings. This could be attributable to persistent inoculation of human hosts in high transmission settings leading to higher parasite densities [41] and concomitant levels of HRP2 antigen reaching the detection limits of both RDTs [42]. In other studies, the difference in the sensitivities of the UsmRDT relative to the conventional RDT was much higher than reported in this study. Differences of 20% -40% have been observed in a high transmission setting of Uganda and a low transmission setting of Myanmar respectively [35]. The slight difference in specificity between the UsmRDT and the mRDT was however similar to a previous report from Uganda and Myanmar [35]. The lower specificity of the UsmRDT could be attributed to the ability of the UsmRDT to detect lower concentration of the HRP2 antigens which persists for extended periods after parasites have been cleared from the blood [20, 21]. The persistence of HRP2 antigen levels in people living in malaria endemic countries, including Ghana is likely due to a frequent and constant exposure to malaria parasites [43], which results in a buildup of HRP2 antigen concentrations that persists over long periods of time [44]. Although the HRP2 antigen has been found to persist for extended periods, the antigen is also known to degrade over time such that HRP2 concentrations maybe present at concentrations that are below the detection limit of standard malaria RDTs but detectable by the UsmRDT. The similar performance of both UsmRDT and mRDT in the pregnant women but higher sensitivity of the UsmRDT than the mRDT in the non-pregnant women only was an interesting finding. This observation could be attributable to pregnant women having placenta that serves as an extra site for harboring parasites [3] and thus allowing the release of additional HRP2 antigen from the parasites sequestered within the placenta. The additional HRP2 antigen results in a concentration that is within the threshold of both UsmRDT and the mRDT. Non-pregnant women on the other hand do not have parasites sequestered in placenta to release additional amounts of HRP2 antigen into circulation, which results in lower HRP2 concentrations, some of which are below the detection limit of the mRDT.

The UsmRDT and the mRDT kits exhibited similar specificities and positive predictive values, however the sensitivity and negative predictive values for the UsmRDT were higher than that of the mRDT kit. The level of agreement between the UsmRDT and PCR was substantial and higher than that for the mRDT kit and PCR, which was moderate [45] most likely due to the UsmRDT having a detection limit closer to PCR than the mRDT. In a study conducted in Uganda and Tanzania, the UsmRDT identified between 20 and 40% more PCR positive samples than the standard RDT [22, 35]. The Tanzania study identified the UsmRDT to also have a slightly higher level of agreement than the standard RDT kit had with ultrasensitive quantitative polymerase chain reaction (qPCR) [22]. Among the pregnant women, both RDTs had the same sensitivity and specificity, similar to results reported by a study in Indonesia [24]. This might be because the amount of antigen released into circulation by the sequestered parasite during pregnancy is enough for detection by both types of RDTs.

The high prevalence of false negative RDT results recorded by both the UsmRDT and the mRDT could have been due to the presence of low parasite densities, below their respective lower limit of detection. Low-density parasites are prevalent in asymptomatic parasite carriers during the off-peak malaria season [46]. The presence of parasites with *pfhrp*2 gene deletions could also result in false negative RDT results [47–49]. However, *pfhrp*2 gene deletions were not determined in this study.

## Conclusion

Although the UsmRDT kit was not as sensitive as PCR at detecting asymptomatic *P. falciparum* carriage, it correctly identified *P. falciparum* in 9.3% of the study participants that were not captured by the mRDT kit. In malaria endemic settings, the UsmRDT would provide an added advantage by identifying more asymptomatic *P. falciparum* carriers than the mRDT kit for the administration of targeted treatment.

## Supplementary information

**Additional file 1.** Data analysis output files.

## Data Availability

All data generated or analysed during this study are included in this published article.

## Abbreviations

PCR: Polymerase Chain Reaction
RDT: Rapid Diagnostic Test
mRDT SD: Bioline Malaria Ag P.f RDT
UsmRDT: Ultra-sensitive Alere™ Malaria Ag P.f RDT
HB: Haemoglobin
